# A Novel Sequencing Method for Quantification of ZIKV RNA in Individual Cells

**DOI:** 10.21769/BioProtoc.5645

**Published:** 2026-03-20

**Authors:** Min Hao, Yisong Wang, Dianyi Du, Wenrong Yang, Qiuzhe Guo, MingJing Tang, Libo Liu, Wei Yang, Yuxuan Liu, Chunyuan Luo, Jing Chen, Peigang Wang, Jing An, Yang Zhou

**Affiliations:** 1 Fuwai Yunnan Hospital, Chinese Academy of Medical Sciences, Affiliated Cardiovascular Hospital of Kunming Medical University, Kunming, China; 2 Yunnan Provincial Cardiovascular Clinical Medical Research Center, Kunming, China; 3 Department of Microbiology, School of Basic Medical Sciences, Capital Medical University, Beijing, China; 4 Fifth Medical Center of PLA General Hospital, Beijing, China; 5 Department of Parasitology, School of Basic Medical Sciences, Guizhou Medical University, Guiyang, Guizhou, China; 6 National Center of Technology Innovation for Animal Model, State Key Laboratory of Respiratory Health and Multimorbidity, Key Laboratory of Pathogen Infection Prevention and Control (Peking Union Medical College), Ministry of Education, NHC Key Laboratory of Comparative Medicine, Chinese Academy of Medical Sciences and Peking Union Medical College, Beijing, China

**Keywords:** Targeted single-cell RNA sequencing, Zika virus, Virus enrichment and host transcriptome libraries, Virus quantification, Mouse brain cell isolation

## Abstract

Single-cell RNA sequencing (scRNA-seq) is a powerful technique for exploring cellular heterogeneity and host–pathogen interactions. This protocol details the Zika virus (ZIKV)-targeted scRNA-seq workflow for preparing high-quality single-cell suspensions from the whole brain tissues of neonatal mice, high-quality single-cell sorting, cDNA reverse transcription, amplification, ZIKV enrichment and host transcriptome library preparation, and sequencing dataset integration in downstream analysis to complete the quantification of ZIKV RNA in individual cells.

Key features

• Preparation of high-quality single-cell suspensions from the whole brain tissues of neonatal mice.

• ZIKV-specific magnetic beads for using the ZIKV and host cell RNA capture.

• ZIKV enrichment and host transcriptome library construction, providing a framework for quantifying viral load within individual cells.

• Integration of viral enrichment and host transcriptomic datasets enables the visualization and quantification of ZIKV at single-cell resolution.

## Graphical overview


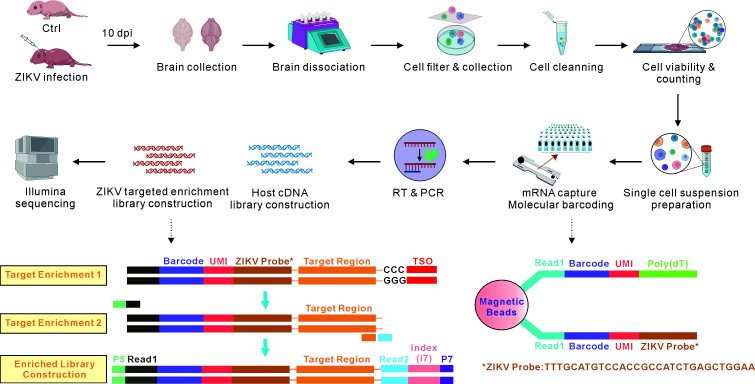


**Working flow of Zika virus (ZIKV)-targeted single-cell RNA sequencing (scRNA-seq).** Brain tissues of ZIKV-infected and uninfected (Ctrl) neonatal mice were collected after 10 days post-infection (dpi). Brain tissues were dissociated with Collagenase type-IV and filtered through a 200-mesh screen. Filtered cells were assessed for viability, density, and aggregation rate to prepare a qualified single-cell suspension. Eligible single cells and ZIKV-modified magnetic beads were loaded into a microfluidic chip to capture host and ZIKV mRNA, respectively. The mRNA underwent reverse transcription for library preparation. Following library quality control, separate host and ZIKV libraries were constructed, enriched, and sequenced on the Illumina platform. After data cleaning and filtering, the host and ZIKV datasets were integrated to analyze the cell characteristics and identify ZIKV-infected target cells.

## Background

The Zika virus (ZIKV) infection seriously challenges global health by leading to neurological disorders, such as the Congenital Zika Syndrome (CZS) in newborns and the Guillain–Barré Syndrome (GBS) in adults. However, the mechanisms of ZIKV–host interaction remain unclear [1]. Single-cell RNA sequencing (scRNA-seq) is a critical tool for exploring cellular heterogeneity and pathogen–host interaction mechanisms at single-cell resolution [2,3]. However, the conventional scRNA-seq relies on poly(dT) primers to capture host RNA with poly(A) tails. ZIKV is an RNA virus that lacks a poly(A) tail, so it cannot be captured by poly(dT). Here, we provide a comprehensive protocol for ZIKV quantification in individual cells using the ZIKV-targeted scRNA-seq.

## Materials and reagents

**Biological materials**

1. SMGC-1 ZIKV strains isolated from a traveler with a recent travel history to Fiji and Samoa, who was identified in Shenzhen, Guangdong Province, China (GenBank No. KX266255)

2. BALB/cAnNCrl neonatal mice (purchased from Beijing Vital River Laboratory Animal Technology Co., Ltd.)

**Reagents**

1. Collagenase type-IV (Sigma, catalog number: C4-BIOC)

2. DNase I (Roche, catalog number: 10104159001)

3. PBS (Thermo Fisher, catalog number: 14190094)

4. AO/PI staining solution (Counter Star, catalog number: 13200066)

5. SCellive^®^ Cell Debris Removal kit (SCellive^®^, catalog number: 13200066)

6. Red blood cell (RBC) lysis buffer (Invitrogen, catalog number: 00-4333-57)

7. FocuSCOPE Single-Cell Multiomics mRNA × ZIKA Virus Detection kit (Singleron Biotechnologies, catalog number: 1002152-5)

*Note: This kit was customized by modifying ZIKV-specific primers (5'-TTTGCATGTCCACCGCCATCTGAGCTGGAA-3') on the capturing beads.*

8. Anhydrous ethanol (Merk, catalog number: 1.00983)

9. PBS-TWEEN^®^ tablets (Merk, catalog number: 524653)

10. Cell lysis buffer (Merk, catalog number: 43-045)

11. QIAcard^TM^ FTA^TM^ wash buffer (Merk, catalog number: WHAWB120204)

**Solutions**

1. Tissue digestion buffer (see Recipes)

2. Debris removal working solution (see Recipes)

**Recipes**

**1. Tissue digestion buffer**

**Table:** 

Reagent	Volume
PBS	320 μL
Collagenase	80 μL
DNase	140 μL

Store at 4 °C.

**2. Debris removal working solution (for brain tissues) [4]**

**Table:** 

Reagent	Volume
Debris removal buffer	2.7 mL
Dilution buffer	0.3 mL

**Laboratory supplies**

1. Scissors (YuYan Instruments, catalog number: Y15103)

2. Finnpipette F3 (Thermo Scientific, catalog numbers: 4640040, 4640050, 4640060)

3. Finntip (Thermo Scientific, catalog numbers: 94052100, 94052200, 94052300, 94052410)

4. Countstar chamber slide (Count Star, catalog number: CO010101)

5. 60 mm × 15 mm tissue-culture treated culture dishes (Corning, catalog number: CLS430166)

6. Cell strainer, 200 screen mesh (Sangon Biotech, catalog number: F513442)

7. 15 mL centrifuge tubes (Corning, catalog number: CLS430791)

8. 1.5 mL microtubes (Corning, catalog number: AXYMCT150CS)

9. 1.5 mL low-binding microcentrifuge tubes (Biopioneer, catalog number: CNT-1.5FL)

## Equipment

1. Digital heating shaking dry bath (Thermo Scientific, catalog number: 88880028)

2. Centrifuge 5920 R (Eppendorf, catalog number: 5948000093)

3. Countstar Rigel S5 (Count Star, catalog number: IN030301)

4. Inverted Microscope BX53 (Olympus, catalog number: BX53F2C)

5. Automated thermal cycler (Applied Biosystems, catalog number: A31491)

6. DynaMag^TM^-2 Magnet (Invitrogen, catalog number: 12321D)

## Software and datasets

1. R software (free download from https://cran.r-project.org/bin/windows/base/) (version 4.3.1, 2023-10-31)

2. RStudio (free download from https://posit.co/download/rstudio-desktop/) (version 2023.03.1 + 446)

3. CeleScope (version 2.0.6, Singleron Biotechnologies) for barcode extraction, UMI counting, and read-level quality control

4. STAR (version 2.6.1a) for read alignment to the mouse reference genome (GRCm38) and the ZIKV reference genome (GenBank accession no. KU321639.1)

5. FeatureCounts (version 2.0.1, subread package) for gene-level quantification

6. Seurat (version v4.1.1) and DoubletFinder (version 2.0.3) R packages for normalization, integration, clustering, and doublet detection

7. MAST (version v1.18.0) R package for the analysis of differentially expressed genes (DEGs)

8. ggplot2 (version 3.4.0) for visualization

## Procedure

**A. Preparation of single-cell suspensions from neonatal mouse whole brain tissues**

**Figure 1.**
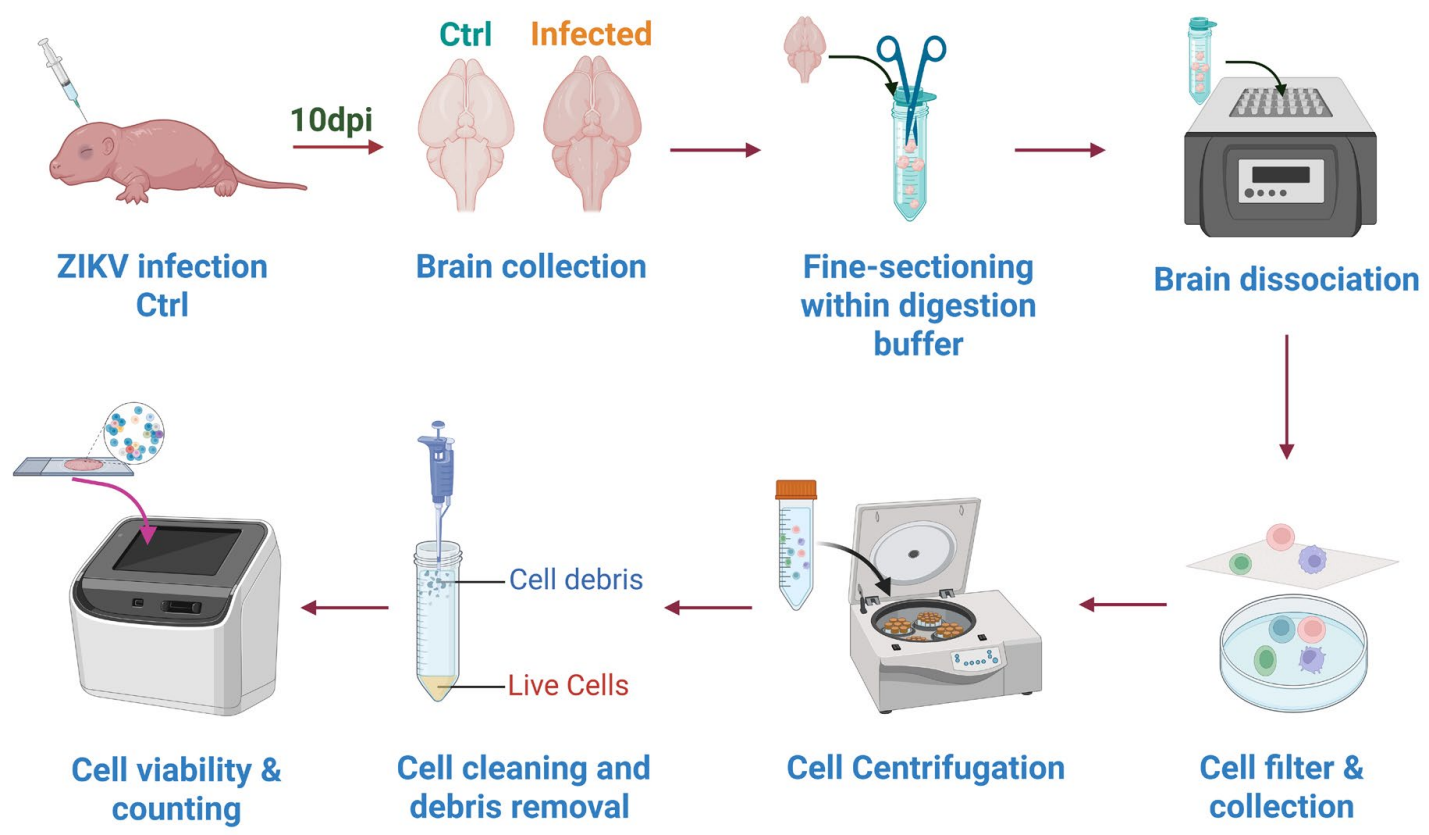
Workflow for preparing the single-cell suspensions. Brain tissues from ZIKV-infected and uninfected (control) neonatal mice (n = 3) at 10 days post-infection (dpi) were collected and digested to cell suspensions. Cell suspensions were filtered using a cell strainer and collected in a culture dish. Filtered cell suspensions were cleaned using the debris removal working solution, and cell viability and counts were evaluated using the automated cell counter.

1. Prepare the tissue digestion buffer (see Recipes).

2. Collect whole brain tissues (brain weight >300–400 mg) from ZIKV-infected and uninfected neonatal mice at 10 days post-infection (dpi). Two-day-old neonatal mice were subjected to intracranial injection for viral infection. The SMGC-1 strain of Zika virus was delivered at a dose of 100 PFU per mouse in a 20 μL volume (Figure 1).

3. Wash the brain tissues with 50 mL autoclaved and ice-cold PBS (4 °C), mince into 1 mm^3^ pieces using scissors, and transfer into the centrifuge tube containing the tissue digestion buffer (Figure 1).

4. Incubate the tube in a dry bath incubator at 37 °C with shaking at 250 rpm for 25 min (Figure 1).

5. Gently pipette the digested brain tissues 5–10 times to dissociate cell aggregates.

6. Place a 200-mesh (70 μm pore size) cell strainer over a culture dish and add an appropriate volume of PBS to submerge the bottom of the dish. Pipette the digested and dissociated brain tissues onto the cell strainer and gently flush the tissues with sterile PBS to allow cells (diameter < 70 μm) to pass through the strainer. Suspend in the culture dish with PBS to prepare the single-cell suspension (Figure 1).

*Note: The volume of cell suspension should be kept under 10 mL.*

7. Collect and transfer the single-cell suspension to a 15 mL centrifuge tube and add PBS to a final volume of 10 mL. Centrifuge at 350× *g* with acceleration 9 and deceleration 9 for 5 min at 4 °C (using Centrifuge 5920 R). Then, discard the supernatant (Figure 1).

8. Add 1 mL of PBS to the 15 mL centrifuge tube to resuspend the cells, then transfer the suspension to a new 15 mL centrifuge tube. Pipette repeatedly to mix gently. Mix 10 μL of the cell suspension with 10 μL of AO/PI staining solution, load the mixture into a cell counting chip, and insert the chip into the cell counter to detect cell viability and aggregation rate of cells.

9. Prepare the debris removal working solution (see Recipes) using the SCellive^®^ Cell Debris Removal kit according to the manufacturer’s instructions [4]. For debris removal, an alternative reagent (e.g., debris removal solution from Miltenyi Biotec, catalog number: 130-109-398) is recommended (Figure 1).

10. Adjust the volume of the cell suspension to 7 mL with pre-chilled PBS, add 3 mL of the prepared debris removal working solution, invert gently to mix, and centrifuge at 450× *g* with acceleration 9 and deceleration 9 for 15 min in a pre-cooled (4 °C) centrifuge.

11. Discard the floating white cell debris from the upper layer of the solution carefully after centrifugation, then slowly discard the remaining supernatant (the viable cells will pellet at the bottom of the tube).

12. Resuspend the viable cell pellet with 1 mL of pre-cooled PBS and transfer to a new 15 mL centrifuge tube. Adjust the volume to 10 mL with pre-cooled PBS, invert to mix, and centrifuge at 300× *g* with acceleration 9 and deceleration 9 for 5 min in a pre-cooled (4 °C) centrifuge. Discard the supernatant to wash off residual debris removal working solution from the surface of the cells.

13. Resuspend the cell pellet with 1 mL of PBS gently and pipette repeatedly to mix. Mix 10 μL of the single-cell suspension with AO/PI staining solution and load into a cell counting chip to evaluate the cell viability and aggregation after using the debris removal working solution.

14. Transfer the single-cell suspension to a new 15 mL centrifuge tube and add PBS to adjust the volume to 4 mL. Then, add RBC lysis buffer at a 1:2 volume ratio (8 mL total). Invert to mix and incubate in a 4 °C refrigerator for 5 min.

15. Centrifuge the incubated single-cell suspension at 300× *g* with acceleration 9 and deceleration 9 for 5 min in a pre-cooled (4 °C) centrifuge. Discard supernatant completely using a pipette after centrifugation.

16. If no RBC clumps are observed in the cell pellet, add 1 mL of PBS to resuspend the pellet and transfer to a new 15 mL centrifuge tube. Adjust the volume of the cell suspension to 10 mL with pre-cooled PBS, invert to mix, and centrifuge again at 300× *g* with acceleration 9 and deceleration 9 for 5 min in a pre-cooled (4 °C) centrifuge. Discard the supernatant completely.

17. Resuspend the cells with 1 mL of pre-cooled PBS, gently pipette to mix, and transfer to a 1.5 mL centrifuge tube. Mix 10 μL of the cell suspension with AO/PI dye for cell counting. A qualified single-cell sample should meet the following criteria: cell viability > 85%, aggregation rate < 10%, and cell concentration of 2.5–3.0 × 10^5^ cells/mL. Keep the qualified single-cell suspension on ice for subsequent use (Figure 1).

**Figure 2.**
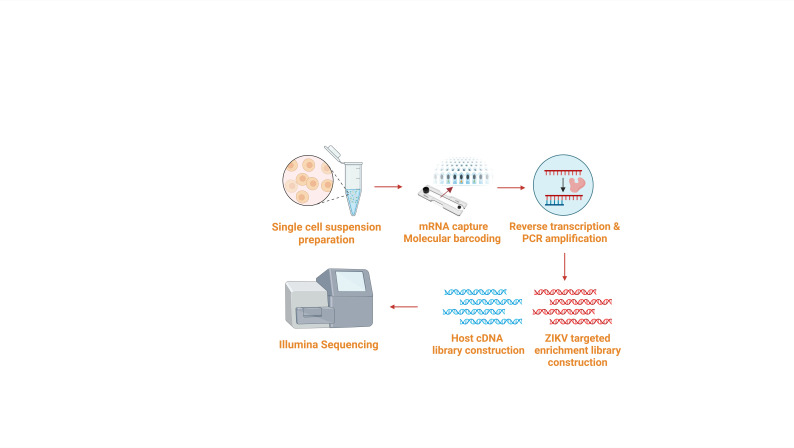
Workflow for single-cell sorting, library construction, and sequencing (sections B–J). Cell suspensions were sorted, and RNA was captured by the microfluidic chip. Reverse transcription was performed to acquire full-length cDNA. The fragmentation enzyme was used for full-length cDNA fragmentation into small fragments. ZIKV-targeted enrichment and host cDNA libraries were constructed. Libraries were sequenced by Illumina sequencing.

**B. Single-cell sorting and mRNA capture (Figure 2)**

The customized ZIKV-targeted scRNA-seq kit was used to perform single-cell sorting according to the manufacturer’s instructions, as follows:

1. Place the microfluidic chip on a clean culture dish. Dispense 200 μL of 100% anhydrous ethanol into the chip through the sample inlet within 10 s. Discard the liquid from the sample outlet and repeat 2–3 times until no air bubbles remain in the chip.

2. Aspirate 200 μL of 0.02% PBST (PBS containing 0.02% Tween-20) and inject into the chip through the inlet within 10 s. Then, discard the liquid from the outlet.

3. Repeat step B2 twice, leave a small amount of liquid at the outlet, cover, and let the cultured dish stand at room temperature for later use.

4. Discard liquid from the inlet and outlet and add 200 μL of PBS to wash the chip (injection time < 10 s). Then, discard excess liquid from the inlet and outlet. Repeat the washing process one more time.

5. Aspirate 100 μL of the resuspended cells and inject into the chip slowly (approximately 30 s); then, discard excess liquid from the outlet immediately.

6. Let the chip stand for 5 min to allow cells to settle into the microwells. During this period, observe cell settlement into the microwells under a 20× microscope.

7. Aspirate 200 μL of PBS and inject it into the chip slowly (approximately 30 s) to wash away excess cells once cells have settled into the microwells. Then, discard the liquid from the inlet and outlet immediately.

8. Repeat step B4 one more time to wash off any cells that have not settled into the microwells.

9. Capture images under a microscope to count the number of cells in each field of view and record the data.

10. Aspirate 60 μL of resuspended ZIKV barcoding beads and add them to the inlet slowly and at a constant speed (over approximately 30 s) to inject the beads into the chip.

11. Aspirate 100 μL of PBS and add into the inlet slowly (approximately 30 s) repeatedly to allow the ZIKV barcoding beads to flow slowly. Then, collect the beads from both the outlet and inlet.

12. Aspirate 200 μL of PBS and inject into the chip slowly (approximately 30 s); then, discard the liquid from the inlet and outlet.

13. Repeat step B12 1–3 times to wash away ZIKV barcoding beads.

14. Observe the settlement of ZIKV barcoding beads into the wells under a microscope.

*Note: Randomly select three fields of view of the microfluidic chip for observation. The bead coverage rate in each field of view must be maintained above 95% under 10× magnification.*

15. Aspirate 100 μL of lysis buffer and inject into the chip slowly through the inlet (approximately 30 s). Then, discard the liquid from both the inlet and outlet immediately.

16. Let the chip stand at room temperature for 5 min to lyse cells, release mRNA, and allow the ZIKV barcoding beads to capture mRNA.

17. Label and place the 1.5 mL centrifuge tube on a 1.5/0.2 mL magnetic rack.

18. Keep the magnetic rack at the bottom of the chip and add 200 μL of wash buffer A to the outlet to wash the surface of the outlet. Then, discard the liquid after washing. Repeat the process twice.

19. Add 200 μL of wash buffer A to the chip outlet. Place the magnetic rack on top of the chip and let it stand for 1 min. With the placement of the magnetic rack, insert a 200 μL pipette tip into the inlet, aspirate 200 μL of liquid, and transfer the collected liquid (which contains the ZIKV barcoding beads) into a pre-cooled 1.5 mL centrifuge tube.

20. Repeat step B19 twice to collect all ZIKV barcoding beads (with captured mRNA) into the same 1.5 mL centrifuge tube.

**C. Reverse transcription (Figure 2)**

1. Prepare the reverse-transcription reaction on ice (Table 1).

**Table 1.** Composition of the reverse transcription reaction mixture

Component	Volume per reaction (μL)
RT master mix	120
TS primer	10
Reverse transcriptase mix	20
RNase inhibitor	5
Nuclease-free water	45
Total	200

*Note. Thaw RT master mix and TS primer at room temperature; gently flick the 2*× *RT master mix to resuspend any precipitate.*

2. Briefly centrifuge the tube containing ZIKV barcoding beads, then place it on a 1.5-mL magnetic stand. Once the suspension clears, carefully aspirate and discard the supernatant without disturbing the beads.

3. Remove the tube from the magnetic stand; add 500 μL of wash buffer B, resuspend, and quick-spin. Return to the magnetic stand and, once clear, aspirate the supernatant.

4. Remove the tube from the magnetic stand, quick-spin, return it to the stand, and aspirate residual liquid with a 20-μL pipette, leaving only the ZIKV barcoding beads at the bottom.

5. Remove the tube from the magnetic stand; add 200 μL of reverse-transcription mix and gently pipette up and down three times.

6. Incubate in a preheated thermomixer at 1,300 rpm for 90 min at 42 °C.

*Note: If processing is delayed after the 42 °C/90 min incubation, heat-inactivate (70 °C, 15 min) and hold at room temperature for ≤15 h (or overnight on the thermomixer/dry block)*.

**D. PCR amplification**

1. Reaction mixture composition (Table 2):

**Table 2.** Composition of the amplification reaction mixture

Component	Volume per reaction (μL)
Amplification master mix	172
A primer mix	32
ZIKV primer mix 1	10
Amplification enzyme	8
Nuclease-free water	178
Total	400

*Note: Thaw amplification master mix, A primer mix, and ZIKV primer mix 1 at room temperature; vortex briefly, quick-spin, then place on ice.*

2. Briefly centrifuge the tube containing the reverse-transcription product and place it on a magnetic stand. Once clear, carefully aspirate the supernatant.

3. Take the tube off the magnetic stand; add 400 μL of PCR master mix, pipette to mix, and aliquot 50 μL into each tube of an 8-tube strip.

4. Cap the 8-tube PCR strip and place it in the thermocycler; amplify using the program in Table 3.

**Table 3.** PCR cycling conditions

Step	Temperature	Time
1	95 °C	0:03:00
2 (cycle = 4)	98 °C	0:00:20
	65 °C	0:00:45
	72 °C	0:03:00
3 (cycle = 10)	98 °C	0:00:20
	67 °C	0:00:20
	72 °C	0:03:00
4	72 °C	0:05:00
5	4 °C	Hold

*Note: After PCR, store samples at 4 °C for up to 72 h or at -20 °C for up to 1 week or proceed directly to the purification of the amplified cDNA*.

**E. Product purification and quality control (QC)**

1. Equilibrate AMPure XP magnetic beads at room temperature for 30 min; vortex thoroughly to resuspend before use.

2. Collect the PCR-amplified product in a 1.5-mL microcentrifuge tube; quick-spin and record the volume. Add 0.6× AMPure XP beads, vortex to mix, incubate for 5 min at room temperature, quick-spin, and then place on a magnetic stand for 5 min. Once clear, aspirate the supernatant into a new 1.5-mL tube and retain.

3. With the tube remaining on the magnetic stand, add 800 μL of freshly prepared 80% ethanol to wash the beads; incubate for 30 s at room temperature and then carefully discard the supernatant.

4. Repeat step E3 one more time (two washes in total).

5. Remove the tube from the magnetic stand, quick-spin, return to the stand, aspirate residual ethanol, and air-dry uncapped for ~2 min (≤5 min).

6. With the tube off the magnetic stand, add 20 μL of buffer EB, resuspend by pipetting, incubate for 5 min at room temperature, quick-spin, and set on the magnetic stand until clear.

7. Collect the clear eluate into a new low-binding microcentrifuge tube to obtain the purified product.

8. Take 1 μL of sample to determine cDNA concentration (>10 ng/μL) and fragment size (200–5000 bp).

*Note: The beads are viscous—vortex thoroughly to fully resuspend before accurately pipetting the specified volume; insufficient mixing may shift the size-selection away from the intended range.*

**F. Construction and sequencing of transcriptome library fragmentation**

1. Pre-program the thermocycler as shown in Table 4, setting the heated lid to 75 °C.

**Table 4.** Thermal program for fragmentation

Step	Temperature	Time
1	4 °C	Hold
2	37 °C	0:05:00
3	65 °C	0:30:00
4	4 °C	Hold

*Note: The initial program verifies proper thermocycler temperature control.*

2. Prepare the reaction mixture as shown below. Gently pipette to mix, quick-spin, and load PCR tubes into the thermocycler, skipping the first program step (Table 5).

**Table 5.** Composition of the fragmentation reaction mixture

Component	Volume per reaction (μL)
Fragmentation buffer	7
Fragmentation enzyme	2
cDNA (QC-passed)	Variable (10 ng/50 ng)
1× TE	Variable
Total	35

*Note: Thaw reagents to room temperature; vortex briefly, then place on ice until use.*

3. Proceed immediately to adapter ligation upon completion of the reaction.

4. After cDNA fragmentation is completed, quality control (QC) is required on the cDNA length. The main peak fragment should be approximately 200–700 bp, and the total library fragment size should be around 500 bp.

**G. Adapter ligation**

1. Pre-program the thermocycler as shown below, with the heated lid disabled (Table 6).

**Table 6.** Thermal program for adapter ligation

Lid temperature		Reaction volume
OFF		70 μL
Step	Temperature	Time
1	4 °C	Hold
2	20 °C	0:15:00
3	4 °C	Hold

2. Ensure the PCR tubes from the previous reaction remain on ice. Then, prepare the following reaction mixture (Table 7):

**Table 7.** Composition of the ligation reaction mixture

Component	Volume (μL)
Fragmented cDNA	35
Ligation mix	30
Ligation booster	1
Adaptor	2.5
Total	68.5

*Note: Thaw reagents to room temperature; vortex briefly, then place on ice until use.*

3. Gently pipette to mix, quick-spin, then load the reaction tube into the thermocycler, skipping the first program step.

**H. Purification of adapter-ligated products**

1. Equilibrate AMPure XP beads to room temperature for 30 min. Quick-spin the product and measure its volume; vortex the beads, add 13.7 μL (0.2×) to 68.5 μL of fragmented product, vortex to mix, and incubate for 5 min at room temperature.

*Note: The beads are viscous—vortex thoroughly to fully resuspend before accurately pipetting the specified volume; insufficient mixing may shift the size-selection away from the intended range.*

2. Quick-spin the PCR tube; place it on a 0.2-mL magnetic stand (DynaMag^TM^-PCR) until clear (~5 min), then carefully aspirate the supernatant into a new sterile PCR tube and retain.

3. With the PCR tube on the magnetic stand, wash the beads with 200 μL of freshly prepared 80% ethanol; incubate for 30 s at room temperature, then aspirate the supernatant.

4. Repeat step H3 one more time (for a total of two washes).

5. Remove the PCR tube from the magnetic stand, quick-spin, return to the stand, aspirate residual ethanol, and air-dry the beads uncapped for 1 min (≤2 min).

6. Remove the PCR tube from the magnetic stand; add 17 μL of 0.1× TE (prepare by diluting 1× TE 1:10 with nuclease-free water), vortex or pipette to resuspend the beads, and incubate for 5 min at room temperature.

7. Quick-spin the PCR tube; place it on the magnetic stand until clear (~5 min), then carefully aspirate 15 μL of the clear supernatant (eluate) into a new sterile PCR tube for PCR enrichment.

**I. PCR enrichment**

1. Keep the PCR tube on ice; prepare the following reaction mixture (Table 8), gently pipette to mix, quick-spin, and load the tube into the thermocycler.

**Table 8.** Composition of the PCR enrichment reaction mixture

Component	Volume (μL)
Purified adapter-ligated product	15
Library Amp Mix V2	25
Indexing primer mix	10
Total	50

*Note: Eight indexing primer mixes are provided; select any one and use one mix per sample.*

2. Pre-program the thermocycler as shown below, setting the heated lid to 105 °C (Table 9).

**Table 9.** PCR cycling conditions for library enrichment

Lid temperature		Reaction volume
105 °C		50 μL
Step	Temperature	Time
1	98 °C	0:00:30
2 (see note for the number of cycles.)	98 °C	0:00:10
	65 °C	0:01:15
3	65 °C	0:05:00
4	4 °C	Hold

*Note: For PCR enrichment, set the cycle number according to the cDNA input: 9 cycles for 50 ng, 12 cycles for 10 ng, and 13 cycles for inputs <10 ng.*

**J. Fragment-size selection of PCR-amplified products**

1. Equilibrate AMPure XP beads to room temperature for 30 min. Quick-spin the PCR tube containing the enriched product and determine its volume; adjust to 50 μL with nuclease-free water if needed. Vortex the beads to resuspend, add 25 μL (0.5× beads-to-sample ratio) to the 50 μL product, vortex to mix, and incubate for 5 min at room temperature.

2. Quick-spin the PCR tube; place it on a 0.2-mL magnetic stand (DynaMag^TM^-PCR) until clear (~5 min). Aspirate the supernatant into a new sterile PCR tube and discard the beads.

3. Vortex to resuspend the beads, add 11.25 μL of AMPure XP beads (0.15× of the sample volume) to the supernatant, vortex to mix, and incubate for 5 min at room temperature.

4. Quick-spin the PCR tube and place it on the magnetic stand until clear (~5 min). Aspirate the clear supernatant into a new PCR tube and retain it.

5. With the PCR tube on the magnetic stand, wash the beads with 200 μL of freshly prepared 80% ethanol; incubate for 30 s at room temperature, then aspirate the supernatant.

6. Repeat step J5 one more time (for a total of two washes).

7. Remove the PCR tube from the magnetic stand, quick-spin, return to the stand, aspirate residual ethanol, and air-dry the beads uncapped for 1 min (≤2 min).

8. Remove the PCR tube from the magnetic stand and add 20 μL of buffer EB to elute. Resuspend the beads by vortexing or pipetting and incubate for 5 min at room temperature.

9. Quick-spin the PCR tube and place it on the magnetic stand until clear (~5 min). Carefully aspirate the supernatant into a new sterile 1.5-mL microcentrifuge tube. Store at -20 °C or -80 °C for up to 1 month.

10. Reserve about 1 μL for library concentration measurement and fragment-size profiling.

**K. Viral gene sequence enrichment**

1. Using 20 ng of QC-passed cDNA, keep the PCR tube on ice and prepare the first-round enrichment PCR mix as detailed below (Table 10); vortex to mix and quick-spin.

**Table 10.** Composition of the first-round enrichment PCR reaction mixture

Component	Volume (μL)
MP master mix	25
ZIKV primer mix 2	25
QC-passed cDNA	Variable
Nuclease-free water	Variable
Total	50

2. Vortex gently to mix thoroughly; load the reaction tube into the thermocycler and run the program as specified in Table 11, with the heated lid set to 105 °C.

**Table 11.** PCR cycling conditions for the first-round viral gene enrichment

Step	Temperature	Time
1	95 °C	0:15:00
2 (cycle = 14)	94 °C	0:00:30
	60 °C	0:01:30
	72 °C	0:01:30
3	72 °C	0:10:00
4	4 °C	Hold

3. Product purification

a. Pre-equilibrate the purification beads to room temperature for 30 min (from 4 °C).

b. Quick-spin the PCR tube and determine the volume. Add 40 μL of AMPure XP beads (0.8× of the product volume), mix by pipetting, incubate for 5 min at room temperature, quick-spin, then place on the magnetic stand for 5 min. Once clear, aspirate the supernatant into a new PCR tube and retain it.

c. With the PCR tube on the magnetic stand, wash the beads twice with 200 μL of freshly prepared 80% ethanol, incubating for 30 s at room temperature for each wash; aspirate the supernatant after each wash.

d. Remove the PCR tube from the magnetic stand, quick-spin, return to the stand, aspirate residual ethanol, and air-dry the beads.

e. Remove the PCR tube from the magnetic stand; add 20 μL of buffer EB, resuspend by pipetting, incubate for 5 min at room temperature, quick-spin, and then place on the magnetic stand until clear.

f. Aspirate the clear eluate into a new low-binding microcentrifuge tube; this is the purified product.

g. Quantify a 1-μL aliquot on a Qubit fluorometer; the product concentration should exceed 1 ng/μL.

**L. Viral gene sequence–enriched library construction**

1. Using 20 ng of the enriched product, keep the PCR tube on ice and prepare the PCR mix for library construction as detailed in Table 12; vortex to mix and quick-spin.

**Table 12.** Composition of the PCR reaction mixture for library construction

Component	Volume (μL)
HiFi PCR PreMix	25
FocuSCOPE adapter mix	1.5
Enriched product (20 ng)	Variable
Nuclease-free water	Variable
Total	50

2. Vortex gently to mix thoroughly; load the reaction tube into the thermocycler and run the program shown below with the heated lid set to 105 °C (Table 13).

**Table 13.** PCR cycling conditions for library construction

Step	Temperature	Time
1	95 °C	0:03:00
2 (cycle = 10)	98 °C	0:00:20
	64 °C	0:00:30
	72 °C	0:01:00
3	72 °C	0:05:00
4	4 °C	Hold

3. Product purification

a. Pre-equilibrate the purification beads to room temperature for 30 min (from 4 °C).

b. Quick-spin the PCR tube and determine the volume. Add 40 μL of purification beads (0.8× of the product volume), mix by pipetting, incubate for 5 min at room temperature, quick-spin, then place on the magnetic stand for 5 min. Once clear, aspirate the supernatant into a new PCR tube and retain it.

c. With the PCR tube on the magnetic stand, wash the beads with 200 μL of freshly prepared 80% ethanol; incubate for 30 seconds at room temperature and then aspirate the supernatant.

d. Repeat step L3c one more time (for a total of two washes).

e. Remove the PCR tube from the magnetic stand, quick-spin, return to the stand, aspirate residual ethanol, and air-dry the beads.

f. Remove the PCR tube from the magnetic stand; add 20 μL of buffer EB and resuspend by pipetting. Incubate for 5 min at room temperature, quick-spin, then return to the magnetic stand until clear.

g. Aspirate the clear supernatant (eluate) and transfer it to a new low-binding microcentrifuge tube; this is the purified product.

4. Quality control of purified amplified products (QC)

a. Quantify a 1-μL aliquot on a Qubit fluorometer.

b. Use a 2-μL aliquot to assess fragment-size distribution.

*Note: The host and ZIKV enrichment libraries were sequenced on a NovaSeq 6000 platform to a depth of 90 Gb using a paired-end 150-base read (PE150) configuration.*

## Data analysis

**A. Data preprocessing and quality control**

1. Process raw FASTQ files using CeleScope v2.0.6 to extract cell barcodes and UMIs from R1, perform read-level quality control (adapter trimming and filtering) with barcode error correction, and forward the corresponding R2 cDNA reads for alignment.

2. Align R2 reads to the hg38 transcriptome using STAR v2.6.1a, retaining only uniquely mapped reads.

3. Count uniquely aligned reads per gene with FeatureCounts v2.0.1, grouping by cell barcode and UMI to generate a gene-expression matrix.

4. For ZIKV-targeted libraries, process with CeleScope v2.0.6 and align R2 reads to the ZIKV reference genome using STAR v2.6.1a (FilterMatchNmin = 80) to ensure high mapping specificity.

5. To discard ambient viral contamination, apply two-step filtering to ZIKV BAM files: first, retain UMIs supported by sufficient read counts; second, retain cells supported by a sufficient number of ZIKV-positive UMIs. Both thresholds are determined using Otsu’s method.

6. Load UMI count matrices from control and ZIKV-infected samples into Seurat. Compute mitochondrial content with PercentageFeatureSet() and retain cells with nFeature_RNA >500 and percent.mt <25%.

**B. Sample normalization, integration, and clustering**

1. Normalize each dataset with NormalizeData (method = "LogNormalize", scale.factor = 10000) and identify highly variable genes using FindVariableFeatures().

2. Scale data with ScaleData() and perform PCA using RunPCA(). The top 50 PCs were selected for downstream analyses (clustering and visualization) based on the elbow plot.

3. To mitigate batch effects, integrate datasets using the CCA-based IntegrateLayers() function.

4. Detect potential doublets with DoubletFinder, optimizing pK via paramSweep() and find.pK(), and retain cells classified as singlets.

5. Using the integrated CCA reduction (reduction = "integrated.cca"), construct an SNN graph with FindNeighbors() on the top 50 PCs, then cluster cells with FindClusters(resolution = 1). Generate UMAP and t-SNE visualizations with RunUMAP() and RunTSNE().

6. Identify cluster markers with FindAllMarkers(), selecting genes expressed in >10% of cells per cluster with log2 fold change >0.25. Visualize clusters and marker expression using Seurat and ggplot2.

**C. Data analysis for precise quantification of ZIKV RNA based on ZIKV-targeted scRNA-seq**

1. Transpose per-cell viral UMI count matrices from control and ZIKV samples. In the control matrix, rename the viral UMI column to “Zika_virus,” in the ZIKV matrix, select the “ZIKV” column and rename it similarly to “Zika_virus.”

2. Append “_1” or “_2” to cell barcodes to ensure unique identifiers across samples.

3. Merge the processed control and ZIKV matrices with rbind(), align the merged matrix with rownames(data@meta.data), and append it to the Seurat metadata with cbind() as a new column named “Zika_virus.”

4. Visualize the per-cell distribution of ZIKV RNA with VlnPlot() and save the plot using ggsave().

## Validation of protocol

This protocol has been used and validated in the following research article(s):

• Zhou et al. [5]. A novel ZIKV-targeted scRNA-seq method for precise quantification of ZIKV RNA. *Journal of Virology* (Figures 4, 6, and 7).

## General notes and troubleshooting

**General notes**

1. The single-cell sorting in this protocol was performed manually, which requires the operator to precisely control the timing of cell and bead injection. Insufficient or excessive duration may lead to suboptimal loading efficiency, thereby compromising the overall experimental outcomes.

2. Although validated by animal models in this protocol, the efficacy of ZIKV-targeted scRNA-seq remains to be established in other species and tissues.

3. This protocol enables ZIKV quantification at the single-cell level and broadens the utility of scRNA-seq via manual preprocessing, particularly valuable for rapid response during outbreaks of infectious diseases.

4. This protocol offers a valuable strategy for rapidly identifying the target cells of RNA viruses at the single-cell level.

**Troubleshooting**

**Problem 1:** How to make sure the beads drop into the wells thoroughly during single-cell sorting?

Solution: If there is a high number of missing beads at the chip inlet, place the recovered ZIKV barcoding beads on a magnetic rack. Aspirate the supernatant, resuspend the beads to a higher concentration, and reload the beads into the areas with bead deficiencies, then let it stand for 10 s before washing. Similarly, if a significant number of unoccupied wells are observed near the outlet, inject the recovered bead suspension into the outlet reservoir. Then, use a pipette to create a gentle backflow from the inlet to redistribute the beads into the unoccupied wells. Then, let the chip stand for 10 s before proceeding to the washing step.

**Problem 2:** Can this protocol be used for other tissues?

Solution: This protocol provides a valuable ZIKV-targeted scRNA-seq strategy for brain tissue. However, due to differences in cellular and tissue architecture, it may not be applicable to other tissue types directly, such as the heart or bone. Therefore, prior to applying this protocol to other tissues, it is essential to conduct preliminary experiments. Specifically, tissue-specific dissociation methods and cell viability assessments must be optimized and validated to ensure protocol compatibility and data quality.

**Problem 3:** How to validate the probe specificity for the targeted scRNA-seq strategy?

Solution: Perform a comprehensive homology analysis of the probe sequence against the NCBI nucleotide database using the BLASTN algorithm. The bioinformatic assessment is the standard practice for predicting potential off-target binding.

**Problem 4:** How to deal with RBC clumps during the preparation of a single-cell suspension?

Solution: Add pre-chilled RBC lysis buffer to the cell pellet at a ratio of 1:3–5 (e.g., 3–5 mL of lysis buffer per 1 mL of cell pellet). Gently pipette the mixture until thoroughly homogenized. Centrifuge at 130–200× *g* for 5 min, then carefully discard the supernatant. Collect the pellet and resuspend it in an appropriate volume of Hank’s solution or serum-free culture medium. Repeat the centrifugation and washing steps 2–3 times to completely remove residual lysis buffer and RBC debris. If lysis is incomplete, repeat the procedure 2–3 times until red blood cells are fully eliminated.

**Problem 5:** What to do if cell quality does not meet one of the criteria (cell viability >85%, aggregation rate <10%, and cell concentration of 2.5–3.0 × 10^5^ cells/mL)?

Solution: If cell viability is <85%, to ensure subsequent data quality and analytical accuracy, we strongly recommend not proceeding to the next step and advise re-collecting the sample for tissue dissociation. If the sample is particularly precious or difficult to obtain, subsequent steps may be attempted when cell viability is ≥70%. If the aggregation rate is >10%, gently pipette the suspension 5–10 times to reduce the aggregation rate. If the cell concentration is below 2.5–3.0 × 10^5^ cells/mL, calculate the current volume of the cell suspension. If the concentration is below 2.5–3.0 × 10^5^ cells/mL, add more of the original cell stock and re-measure the concentration; if it is above 2.5–3.0 × 10^5^ cells/mL, dilute with PBS and then re-measure the concentration.

**Problem 6:** What to do if the yield is significantly lower than expected (<10ng total) in section E?

Solution: First, ensure that the beads are fully equilibrated at room temperature and vortexed until completely resuspended before use, as inconsistent bead suspension can reduce capture efficiency. Second, assess the concentration and integrity of the PCR product prior to purification; poor-quality or degraded input will inevitably lower final recovery. If the fragment distribution is skewed toward shorter fragments, consider adjusting the beadtosample ratio (e.g., 0.6–0.7×) and optimize the number of PCR cycles to avoid overamplification and accumulation of short products. During the elution step, strictly control the drying time (<5min) and ensure thorough resuspension and adequate incubation of the beads in the elution buffer to improve elution efficiency. Additionally, always use freshly prepared 80% ethanol for washing to prevent contamination and reduced washing performance. If the yield remains suboptimal after these adjustments, reevaluate the initial sample quality or repeat the library preparation procedure.

**Problem 7:** What to do if the product concentration is <1 ng/µL after library construction?

Solution: The primary diagnostic step involves assessing the library fragment distribution using a Bioanalyzer or Fragment Analyzer. If the electrophoretic profile displays an appropriate size distribution but low signal intensity, this indicates insufficient yield of amplifiable library molecules. In such cases, the library may be re-amplified by supplementing 2–4 additional PCR cycles while strictly avoiding over-amplification. Should a prominent peak appear in the range of 120–150 bp, suggesting contamination by adapter or primer dimers, the library should be re-purified using an increased ratio of SPRI beads (e.g., 0.8–0.9×) to selectively remove short-fragment contaminants. If the profile lacks a distinct library peak or exhibits an abnormal pattern, this implies potential failure in early library preparation steps—such as fragmentation, end repair, A-tailing, or adapter ligation—necessitating library reconstruction. In this scenario, particular attention must be paid to key factors including input nucleic acid quality, reagent activity, and purification efficiency. Following any corrective intervention, the library must be re-quantified using both spectrophotometric (e.g., Qubit) and qPCR methods, and fragment distribution must be re-verified to ensure compliance with platform-specific requirements before pooling and sequencing.
